# Degradation of Cylindrospermopsin Spiked in Natural Water (Paranoá Lake, Brasília, Brazil) by Fenton Process: A Bench–Scale Study

**DOI:** 10.3390/toxins16120536

**Published:** 2024-12-12

**Authors:** Matheus Almeida Ferreira, Cristina Celia Silveira Brandão, Yovanka Pérez Ginoris

**Affiliations:** Environmental Technology and Water Resources Postgraduate Program, Department of Civil and Environmental Engineering, University of Brasília, Brasília 70910-900, Brazil; yovanka@unb.br

**Keywords:** advanced oxidation process, Fenton process, cyanotoxins removal, cylindrospermopsin, Paranoá lake

## Abstract

The frequency and intensity of harmful cyanobacterial blooms have increased in the last decades, posing a risk to public health since conventional water treatments do not effectively remove extracellular cyanotoxins. Consequently, advanced technologies such as the Fenton process are required to ensure water safety. The cyanotoxin cylindrospermopsin (CYN) demands special attention, as it is abundant in the extracellular fraction and has a high toxicological potential. Hence, this study aimed to assess the application of the Fenton process for the oxidation of CYN spiked in natural water from Paranoá Lake (Brasília, Brazil). The H_2_O_2_/Fe(II) molar ratio was evaluated from 0.2 to 3.4, with an optimum molar ratio of 0.4, achieving a CYN degradation efficiency of 97.8% when using 100 µM of H_2_O_2_ and 250 µM of Fe(II). The CYN degradation efficiency, using 75 µM of H_2_O_2_ and 187.5 µM of Fe(II), decreased by increasing the initial pH (from 96.2% at pH 2 to 23.0% at pH 9) and the initial CYN concentration (from 93.7% at 0.05 µM of CYN to 85.0% at 0.2 µM of CYN). At the optimum H_2_O_2_/Fe(II) molar ratio of 0.4, the hydroxy radical scavengers tested (124.3 µM C of algogenic organic matter, 5 mg L^−1^ of humic acid, and 513.3 µM of methanol) did not considerably affect the CYN degradation, reaching a maximum CYN degradation reduction from 98.3% to 82.2%.

## 1. Introduction

The anthropogenic nutrient enrichment of surface freshwater associated with higher Earth temperatures in recent decades tends to increase the frequency and intensity of cyanobacterial blooms [1]. The potential health risks posed by cyanobacteria blooms in drinking water supplies are a growing concern, since dozens of cyanobacteria species can produce taste and odor compounds and various toxins. Aside from the well-studied microcystin, the cyanotoxin cylindrospermopsin (CYN) has been gaining increasing attention because CYN–producing species are found in aquatic systems worldwide, including Brazil [2,3]. Additionally, it is known that CYN is harmful to human health, causing primarily liver and kidney damage [4,5,6]. Thus, regarding CYN in drinking water, the World Health Organization recommended guideline values of 0.7 µg L^−1^ and 3.0 µg L^−1^ for lifetime and short–term exposure, respectively [7]. In Brazil, the guideline value of 1 µg L^−1^ for CYN in drinking water, established by the Ministry of Health, became mandatory only in 2021 [8] due to the growing number of reports regarding CYN occurrences in the country.

In general, significant concentrations of cyanotoxins such as microcystins, nodularins, anatoxins, and saxitoxins are found in natural aquatic environments when cell lysis occurs, except for CYN, which can also be released from viable cells during its entire life cycle [9]. Since conventional water treatment processes do not effectively remove water–soluble extracellular cyanotoxins like CYN [10,11,12,13,14], and extracellular CYN tends to be relatively stable in surface water [15], the use of advanced water treatment processes is necessary to remove CYN in drinking water treatment to produce safe water.

Several advanced processes are commonly employed in drinking water treatment to remove dissolved cyanotoxins, including activated carbon adsorption and membrane separation [16,17,18,19]. Although these methods effectively remove toxins from the aqueous phase, they transfer the contaminants to another phase, adsorbed onto activated carbon or concentrated in the membrane retentate, requiring further treatments before disposal.

On the other hand, advanced oxidation processes (AOPs) offer the advantage of degrading pollutants into less harmful or inert compounds, which reduces the necessity for secondary waste treatment. Various AOPs have been successfully applied to remove CYN from water, including UV combined with $S_{2}O_{8}^{2-}$, ${HSO}_{5}^{-}$, or $H_{2}O_{2}$ [20,21,22], ${TiO}_{2}$ photocatalysis [23], ${TiO}_{2}$ assisted ozonation [24], electrochemical oxidation [25], non-thermal plasma [26], Fenton or Fenton–like [27,28,29,30], and photo–Fenton [31]. Most of these studies were conducted using ultrapure water.

Amongst AOPs, the Fenton process has received attention for its simplicity, cost-effectiveness, high performance, and the non–toxic nature of the reagents H_2_O_2_ and Fe(II) [14,32,33,34].The primary oxidizing agent, the hydroxyl radical (OH•), is generated from reactions between H_2_O_2_ and Fe(II) and can react with a wide variety of organic and inorganic compounds. The efficiency of this process depends on parameters including temperature, pH, initial concentration of the target pollutant, H_2_O_2_ and Fe(II) dosages, and H_2_O_2_/Fe(II) molar ratio.

Despite the operational simplicity of the Fenton process, which is attractive to small communities and developing countries such as Brazil, and the high degradation efficiencies of CYN in ultrapure water matrices, up to 100%, as reported by Ferreira et al. [27] and Schneider et al. [28], to the best of our knowledge, no previously published study has focused on applying the Fenton process to degrade CYN in natural waters. This study is a continuation of the work by Ferreira et al. [27], conducted by the same group of authors, and aims to expand on those findings by exploring the applicability of the Fenton process in natural water matrices. Thus, the objective of this bench scale study was to evaluate the oxidation of CYN spiked in Paranoá Lake water (Brasília, Federal District, Brazil) by the Fenton process, with emphasis on the effect of H_2_O_2_/Fe(II) molar ratio, H_2_O_2_ and Fe(II) dosages, initial CYN concentration, initial pH, and OH• scavengers including algogenic organic matter (AOM), humic acid (HA), and methanol on the CYN degradation efficiency.

## 2. Results and Discussion

### 2.1. The Effect of H_2_O_2_/Fe(II) Molar Ratio on CYN Degradation

**Figure 1:**
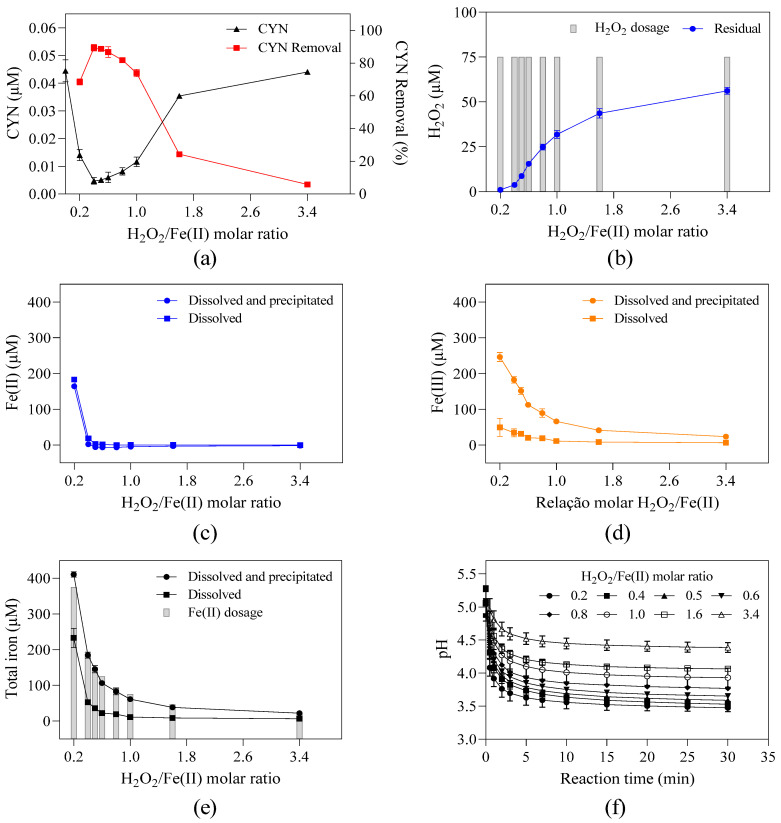
Residual concentration and/or removal of (**a**) CYN, (**b**) H_2_O_2_, (**c**) dissolved and total fractions of Fe(II), (**d**) dissolved and total fractions of Fe(III), (**e**) dissolved and total fractions of total iron, and (**f**) pH–time profile during Fenton oxidation for various H_2_O_2_/Fe(II) molar ratios. H_2_O_2_ dosage fixed at 75 µM and Fe(II) from 22.1 to 375.0 µM. Error bars represent the standard deviation of the mean based on three replicates.

The effect of the H_2_O_2_/Fe(II) molar ratio on CYN degradation was evaluated in a range of 0.2 to 3.4 by fixing the H_2_O_2_ dosage at 75 µM and varying the Fe(II) dosage from 22.1 to 375.0 µM (Figure 1). The appropriate H_2_O_2_/Fe(II) molar ratio enhances Fenton process efficiency and depends not only on experimental conditions, but also on the type and concentration of the target pollutant.

The degradation efficiency of CYN increased from 68.4% to 89.3% (the highest CYN degradation) when the H_2_O_2_/Fe(II) molar ratio increased from 0.2 to 0.4, and then decreased rapidly from 88.6% to 20.5% when the H_2_O_2_/Fe(II) molar ratio increased from 0.5 to 1.6 followed by a slight decrease, achieving about 1.0% at the H_2_O_2_/Fe(II) molar ratio of 3.4 (Figure 1a). Ferreira et al. [27] reported a similar trend in which the highest CYN degradation efficiency of 81% was achieved using the H_2_O_2_/Fe(II) molar ratio of 0.4 under the following experimental conditions: 0.05 µM of CYN diluted in ultrapure water, 25 µM of H_2_O_2_, 62.5 µM of Fe(II), pH 5.0, and 30 min reaction time. Similarly, Schneider et al. [28] evaluated the degradation of 0.72 µM of CYN diluted in ultrapure water at pH 3.0 and obtained an optimum H_2_O_2_/Fe(II) molar ratio of 0.5 with a degradation efficiency of about 31% after a 60 min reaction using 10 µM of H_2_O_2_ and 20 µM of Fe(II).

In the absence of H_2_O_2_ and Fe(II), no degradation of CYN was observed after the 30 min reaction period.

When comparing the CYN degradation efficiency at the H_2_O_2_/Fe(II) molar ratio of 0.2 and 0.4, the lower degradation obtained using the H_2_O_2_/Fe(II) molar ratio of 0.2 was probably caused by the excess Fe(II) (Figure 1c), as the residual H_2_O_2_ was similar for both H_2_O_2_/Fe(II) molar ratios (Figure 1b). The excess Fe(II) can increase the OH• consumption (Equation (1)), thereby diminishing the overall degradation of CYN.

(1) OH•+Fe(II)→Fe(III)+OH−

For H_2_O_2_/Fe(II) molar ratios between 0.4 and 3.4 (Figure 1a), the observed reduction in CYN degradation efficiency caused by increasing H_2_O_2_/Fe(II) molar ratio may be explained by the combination of three main factors.

The first factor is the amount of OH• generated. Since the H_2_O_2_ dosage was fixed, the Fe(II) dosage decreases as the H_2_O_2_/Fe(II) molar ratio increases, which resulted in a higher residual H_2_O_2_ as observed in Figure 1b, thus indicating a lower OH• generation.

The second factor is the H_2_O_2_ and Fe(II) scavenging activity (Equations (1) and (2)). While the residual Fe(II) remained nearly constant, the progressive increase in residual H_2_O_2_ led to a higher OH• consumption, resulting in a decrease of CYN degradation efficiency.

(2) OH•+H2O2→HO2•+H2O

The third factor is the pH of the study water. As higher degradation efficiencies by the Fenton process are typically achieved under acidic conditions, around pH 3 [35,36,37], and the hydrolysis of Fe(III) produced in the Fenton reaction contributes to the acidification of water [38], the reduction in Fe(II) dosage caused by increasing the H_2_O_2_/Fe(II) molar ratio may have led to a higher final pH (Figure 1f), thereby reducing the degradation efficiency.

In addition, due to the turbidity of the study water, the higher precipitation of Fe(III) at lower H_2_O_2_/Fe(II) molar ratios (Figure 1d) can remove natural organic matter by coagulation–flocculation, thereby reducing competition for the oxidizing agents.

It must be emphasized that the high residual H_2_O_2_ concentrations observed at high H_2_O_2_/Fe(II) molar ratios indicate that higher degradation efficiency of CYN could be achieved with longer reaction times, as the generation of Fe(II) can be accomplished by residual H_2_O_2_ (Equation (3)).

(3) $$Fe(III)+H_{2}O_{2}\to Fe(II)+{HO}_{2}^{•}+H^{+}$$

### 2.2. The Effect of H_2_O_2_ and Fe(II) Dosages on CYN Degradation at a Fixed H_2_O_2_/Fe(II) Molar Ratio

**Figure 2:**
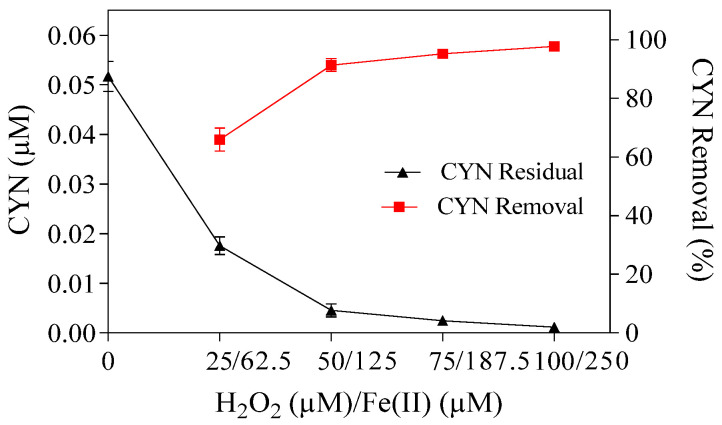
Residual concentration and removal of CYN by the Fenton process for different H_2_O_2_ and Fe(II) dosages at the H_2_O_2_/Fe(II) molar ratio of 0.4. Initial pH of about 5.0, and 30 min reaction. Error bars represent the standard deviation of the mean based on three replicates.

The effect of Fenton reagent dosages on CYN degradation was evaluated at the optimum H_2_O_2_/Fe(II) molar ratio of 0.4, with H_2_O_2_ and Fe(II) dosages ranging from 25 to 100 µM and 62.5 to 250 µM, respectively (Figure 2).

As shown in Figure 2, for a fixed H_2_O_2_/Fe(II) molar ratio, the CYN degradation efficiency increases as the H_2_O_2_ and Fe(II) dosages increase until the reagents reach a specific dosage, above which any increase in the degradation efficiency is marginal. Specifically, the CYN degradation efficiency was 66% with 25 µM H_2_O_2_ and 62.5 µM Fe(II), 91.3% with 50 µM H_2_O_2_ and 125 µM Fe(II), 95.2% with 75 µM H_2_O_2_ and 187.5 µM Fe(II), and 97.8% with 100 µM H_2_O_2_ and 250 µM Fe(II). A similar trend was reported by Schneider et al. [28] and Ferreira et al. [27], who evaluated the CYN degradation by the Fenton process in ultrapure water.

Table 1 presents a comparison of the results of CYN degradation using Paranoá Lake water as matrix with a previous work using ultrapure water as matrix [27].

The natural organic matter in Paranoá Lake water can hinder CYN degradation efficiency. Using 25 µM H_2_O_2_ and 62.5 µM Fe(II), the CYN degradation decreased from 81% in ultrapure water to 66% in Paranoá Lake water (Table 1).

However, despite the natural organic matter in Paranoá Lake water, a similar CYN degradation of about 91% was reported by Ferreira et al. [27] and observed herein when using 50 µM H_2_O_2_ and 125 µM Fe(II), which may be attributed to the Fe(II) scavenging effect observed by Ferreira et al. [27]. For a fixed H_2_O_2_/Fe(II) molar ratio, the authors reported that the residual Fe(II) decreased when the H_2_O_2_ and Fe(II) dosages increased, suggesting a higher Fe(II) scavenging activity (Equation (1)), which was not observed in this study, as the residual Fe(II) was similar for all H_2_O_2_ and Fe(II) dosages tested (Supplementary Figure S1b). Other Fenton reagent residuals and the pH–time profile of CYN degradation are shown in Supplementary Figure S1.

It is worth mentioning that even at the highest H_2_O_2_ and Fe(II) dosages used in this study, less than 5% degradation of CYN was detected when H_2_O_2_ and Fe(II) were tested separately, that is, 100 µM of H_2_O_2_ alone and 375.0 µM of Fe(II) alone—similarly, Schneider et al. [28], Ferreira et al. [27], and Munoz et al. [29] reported no considerable CYN degradation by the Fenton and Fenton–like processes testing Fenton reagents separately.

### 2.3. The Effect of Initial CYN Concentration on Fenton’s Efficiency at a Fixed H_2_O_2_/Fe(II) Molar Ratio

**Figure 3:**
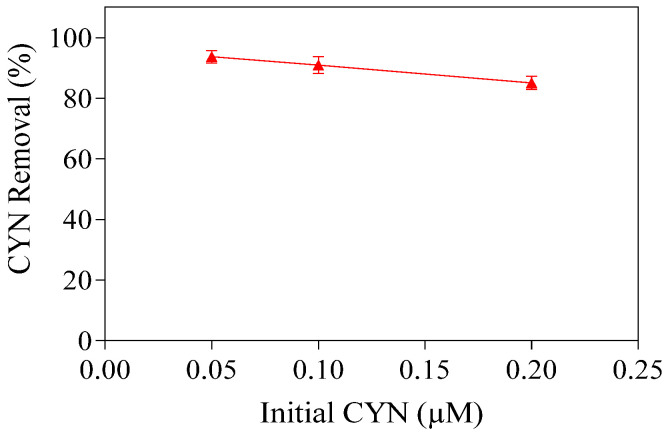
Removal of CYN for various initial CYN concentrations. Initial pH about 5.0, 75 µM of H_2_O_2_, 187.5 µM of Fe(II), and 30 min reaction. Error bars represent the standard deviation of the mean based on three replicates.

At the H_2_O_2_/Fe(II) molar ratio of 0.4, the CYN degradation efficiency declines slightly as the initial CYN concentration increases (Figure 3). The Fenton reagent residuals and the pH–time profile of CYN degradation are shown in Supplementary Figure S2.

When the initial CYN concentration was 0.05, 0.1, and 0.2 µM, the degradation efficiency after the 30 min reaction was 93.7%, 90.9%, and 85.0%, respectively. A similar trend was reported by Park et al. [39], who applied the Fenton process for MC–LR degradation in ultrapure water with an initial pH of 5.2 and using 147 µM H_2_O_2_ and 90 µM Fe(II). Under such conditions, the authors obtained 92%, 80%, and 77% degradation efficiency when the initial MC–LR concentrations were 0.002, 0.020, and 0.200 µM, respectively. Al Momani et al. [40] also observed a similar behavior regarding MC–LR degradation by the Fenton process using 0.15 µM H_2_O_2_ and 0.90 µM Fe(II), with about 97% degradation for an initial concentration of 0.5 µM MC–LR and 75% for an initial concentration of 1.0 µM MC–LR.

It must be noted that the increase in initial CYN concentration resulted in a proportional increase in methanol concentration in the study water. When initial CYN concentrations were 0.05, 0.1, and 0.2 µM, the methanol concentrations in the study water were 513.3, 1026.6, and 2053.2 µM, respectively. The change in CYN degradation efficiency from 93.7% to 85.0% with the increase in methanol concentration from 513.3 µM to 2053.2 µM indicates that the interference of methanol on CYN degradation is limited under the conditions evaluated. A detailed discussion of the interference of methanol on CYN degradation can be found in Section 2.6.

### 2.4. The Effect of Initial pH on CYN Degradation at a Fixed H_2_O_2_/Fe(II) Molar Ratio

**Figure 4:**
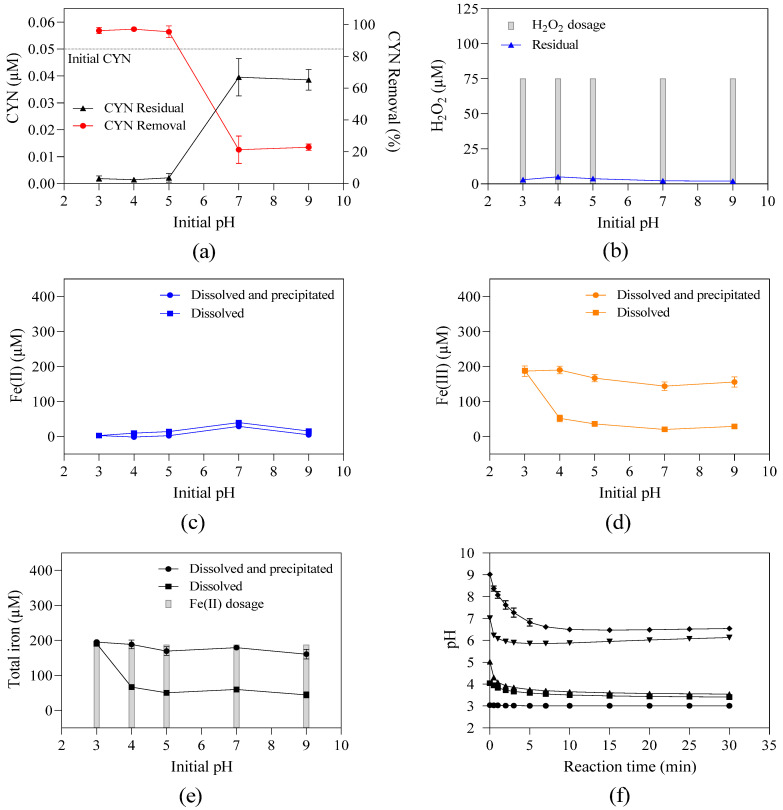
Residual concentration and/or removal of (**a**) CYN, (**b**) H_2_O_2_, (**c**) dissolved and total fractions of Fe(II), (**d**) dissolved and total fractions of Fe(III), (**e**) dissolved and total fractions of total iron, and (**f**) pH–time profile during Fenton oxidation for various initial pH values. H_2_O_2_ dosage of 75 µM and Fe(II) of 187.5 µM. Error bars represent the standard deviation of the mean based on three replicates.

As is well known, pH is essential to Fenton’s efficiency. To evaluate the effect of the initial pH on CYN degradation, experiments were conducted with a fixed H_2_O_2_/Fe(II) molar ratio of 0.4 at different initial pH values ranging from 3 to 9, as shown in Figure 4f. After the initial Fenton reactions, the pH value dropped rapidly during the first 5 min and remained virtually unchanged. After a 30 min reaction, the pH value decreased from 9.0 to 6.5, from 7.0 to 6.1, from 5.0 to 3.5, and from 4.0 to 3.4, and there was no change when the initial pH was 3.0.

The CYN degradation efficiency tends to decrease as the initial pH increases. When the initial pH value was 3, 4, 5, 7, and 9, the CYN degradation efficiency was 96.2%, 97.2%, 95.7%, 21.0%, and 23.0%, respectively.

An increase in pH results in the enhancement of both Fe(III) precipitation [41] and H_2_O_2_ decomposition [42,43], thereby reducing the efficiency of the Fenton process.

Under the conditions herein evaluated, the effect of Fe(III) precipitation can be neglected probably due to the H_2_O_2_/Fe(II) molar ratio of 0.4, with an excess Fe(II), which may indicate no need for Fe(III) dissolved available for Fe(II) regeneration (Equation (3)). According to De Laat and Gallard [41], Fe(III) precipitation occurs at pH values above 3.2, as also observed in Figure 4d.

Under acidic conditions, despite the differences in the initial pH and the amount of Fe(III) precipitated at pH 3 (lower than the limit of detection) and pH 4 and 5 (130.8 to 138.5 µM), as observed in Figure 4d, the final pH remained within the optimal pH range for the Fenton process, around pH 3 (Figure 4f), which can explain the similar CYN degradation efficiency obtained for this pH range. Additionally, there were no considerable differences in the Fe(III) precipitation (123.9 to 138.5 µM) among the initial pH values of 4, 5, 7, and 9 (Figure 4f). Thus, the decreased CYN degradation efficiency caused by increasing pH can be attributed to the H_2_O_2_ decomposition, reducing the generation of OH•, and the alkalinity found in Paranoá Lake water (28.8 mg/L of CaCO_3_) as bicarbonate and carbonate ions can likely react with OH•.

A similar effect was reported by Schneider et al. [28], who evaluated the degradation of CYN in ultrapure water by the Fenton process (concentrations of 0.72 μM of CYN, 10 μM of H_2_O_2_, 20 μM of Fe(II) and 60 min of reaction) and reported degradations of CYN of about 12%, 58%, 50%, and 2% for the respective initial pH values of 3.0, 4.3, 5.6, and 11.0. Regarding MC–LR degradation in ultrapure water by the Fenton process, Zhong et al. [35] and Park et al. [39] also observed a similar behavior.

### 2.5. The Effect of Humic Acid (AH) and Algogenic Organic Matter (AOM) on CYN Degradation at a Fixed H_2_O_2_/Fe(II) Molar Ratio

**Figure 5:**
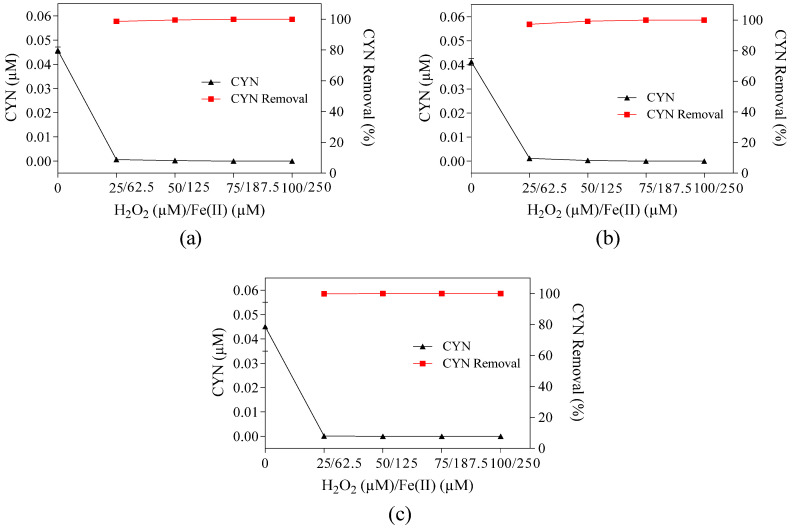
Removal and residual concentrations of CYN for different H_2_O_2_ and Fe(II) dosages at H_2_O_2_ Fe(II) of 0.4 in (**a**) ultrapure water, (**b**) ultrapure water containing 5.0 mg/L of HA, and (**c**) ultrapure water containing AOM (NPOC of 125 µM C). Initial pH around 5.0 and 30 min reaction. Error bars represent the standard deviation of the mean based on three replicates.

To evaluate the effect of HA and AOM on CYN degradation, three subsets of experiments were performed in ultrapure water with (I) 0.05 µM of CYN, (II) 0.05 µM of CYN and 5.0 mg/L of HA, and (III) 0.05 µM of CYN spiked with *Raphidiopsis raciborskii* crude extract containing AOM (Figure 5).

As observed in Figure 5, all experiments were conducted at a fixed molar ratio of 0.4 with different H_2_O_2_ and Fe(II) dosages ranging from 25 to 100 µM and 62.5 to 250 µM, respectively. It must be emphasized that the subsets of experiments (I) and (II) were conducted with study water containing 513.3 µM of methanol, while the subset (III) was conducted without methanol.

The CYN degradation in ultrapure water by the Fenton process was 98.5% with 25 µM H_2_O_2_ and 62.5 µM Fe(II), 99.5% with 50 µM H_2_O_2_ and 125 µM Fe(II), and approximately 100% for the other concentrations of 75 and 100 µM H_2_O_2_ and 125, 187.5, and 250 µM Fe(II), respectively.

As expected, adding 5.0 mg/L of HA in ultrapure water caused a slight reduction in CYN degradation efficiency. The CYN degradation in the presence of 5 mg/L of HA was 97.2% with 25 µM H_2_O_2_ and 62.5 µM Fe(II), 99.3% with 50 µM H_2_O_2_ and 125 µM Fe(II), and approximately 100% for the other higher H_2_O_2_ and Fe(II) concentrations tested.

Concerning the effect of the AOM on CYN degradation in ultrapure water, 99.8% of CYN was degraded using 25 µM H_2_O_2_ and 62.5 µM Fe(II), and approximately 100% was degraded using the other higher H_2_O_2_ and Fe(II) concentrations tested.

For details about Fenton reagent residuals and the pH–time profile of CYN degradation, see Supplementary Figures S3–S5.

Despite the reaction rate constants between OH• and methanol, 1.2 to 10.3 × 10^8^ M^−1^ s^−1^ [44,45], between OH• and HA, 5.7 to 6.4 × 10^8^ M^−1^ s^−1^ [46], and between OH• and AOM, 4.0 to 8.0 × 10^8^ M^−1^ s^−1^ [47], have similar orders of magnitude, the different concentrations of these compounds did not considerably affect CYN degradation.

Under the conditions evaluated and with excess H_2_O_2_ (25 to 100 µM) and Fe(II) (62.5 to 250 µM), there were no notable interferences of methanol, HA, and AOM on the degradation of CYN by the Fenton process, as observed in Figure 5. Regardless of the dosages of H_2_O_2_ and Fe(II) and the presence of methanol, HA, or AOM, the CYN degradation efficiency by the Fenton process was higher than 97.2%.

Although this study was conducted under bench–scale conditions, the results herein highlight the potential of the Fenton process for treating natural waters contaminated with CYN. The degradation efficiency observed in this study, under optimized conditions, was higher than 90% in both ultrapure and natural water matrices, even in the presence of NOM, AOM, and other OH• scavengers. This demonstrates the robustness of the Fenton process under conditions that more closely resemble real–world scenarios.

It must be pointed out that the Fenton process can be integrated into rapid mixing units of existing conventional water treatment plants, reducing the need for significant infrastructural modifications while providing efficient oxidative treatment. This is especially relevant for developing countries, where its simplicity, cost-effectiveness, and reliance on non–toxic reagents such as H_2_O_2_ and Fe(II) make it a viable option for improving drinking water quality.

However, implementing the Fenton process must address the challenges of managing residual iron in treated water and iron sludge, as these may pose operational and environmental concerns. These aspects demand further investigation to ensure the process aligns with regulatory standards and minimizes secondary waste generation.

### 2.6. The Interference of Methanol on CYN Degradation

**Figure 6:**
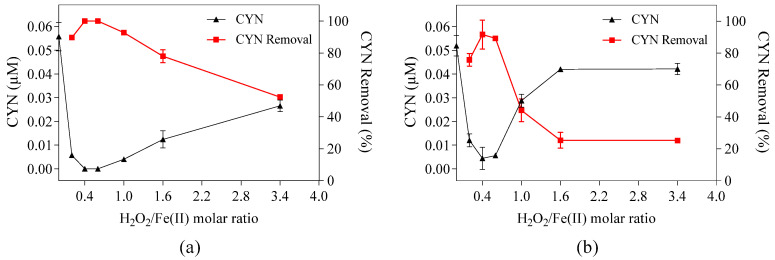
Residual concentration and removal of CYN by the Fenton process for various H_2_O_2_/Fe(II) molar ratios in (**a**) methanol–free ultrapure water and (**b**) ultrapure water containing 513.3 µM of methanol. Initial pH about 5.0, 25 µM of H_2_O_2_, 7.4 to 125 µM Fe(II), and 30 min reaction. Error bars represent the standard deviation of the mean based on two replicates.

One of the CYN stock solutions used for spiking the study water was prepared by dissolving 1.2 mmol of CYN standard in 1 mL of methanol to ultrapure water solution (1:1 *v*/*v*). Thus, when preparing study water with an initial CYN concentration of 0.05 µM, the methanol concentration in this same study water was approximately 513.3 µM. To evaluate the interference of methanol on CYN degradation, the sixth set of experiments was conducted in ultrapure water with and without methanol (Figure 6).

It can be observed in Figure 6 that, regardless of the presence of methanol, the effect of the H_2_O_2_/Fe(II) molar ratio on CYN degradation in ultrapure water presents a similar behavior, in which the degradation efficiency of CYN increased when the H_2_O_2_/Fe(II) molar ratio increased from 0.2 to 0.4–0.6, and then systematically decreased when the H_2_O_2_/Fe(II) molar ratio increased to 3.4.

For the methanol–free ultrapure water (Figure 6a) and the ultrapure water containing 513.3 µM of methanol (Figure 6b), the optimum H_2_O_2_/Fe(II) molar ratio was, respectively, 0.4–0.6 with approximately 100% of CYN degradation, and 0.4 with 91.4% of CYN degradation, indicating that the CYN degradation efficiency was not considerably affected by the presence of methanol at H_2_O_2_/Fe(II) molar ratios smaller than 1.0, including the optimum value of 0.4.

However, for H_2_O_2_/Fe(II) molar ratios higher or equal to 1.0, a substantial reduction in CYN degradation efficiency was observed, achieving maximum CYN degradation reduction from 77.7% in methanol–free ultrapure water to 19.0% in ultrapure water containing 513.3 µM of methanol at the H_2_O_2_/Fe(II) molar ratio of 1.6. The reduction of CYN degradation efficiency caused by increasing the H_2_O_2_/Fe(II) molar ratio can probably be attributed to a lower production of OH• due to lower Fe(II) dosages.

Additionally, CYN has a higher reaction rate constant with OH•, 5.1 to 5.5 × 10^9^ M^−1^ s^−1^ [48,49], than methanol with OH•, 5.7 to 6.4 × 10^8^ M^−1^ s^−1^ [44,45], which may explain, to some extent, the slight influence of methanol on CYN degradation herein observed.

It should be pointed out that the higher CYN degradation efficiencies observed at the H_2_O_2_/Fe(II) molar ratio of 0.4–0.6 indicate an optimal CYN degradation region around the H_2_O_2_/Fe(II) molar ratio of 0.5, which is the stoichiometric value for the overall Fenton reaction (Equation (4)).

(4) $$2Fe\left(II\right)+H_{2}O_{2}+{2H}^{+}\to 2Fe(III)+2H_{2}O$$

**Figure 7:**
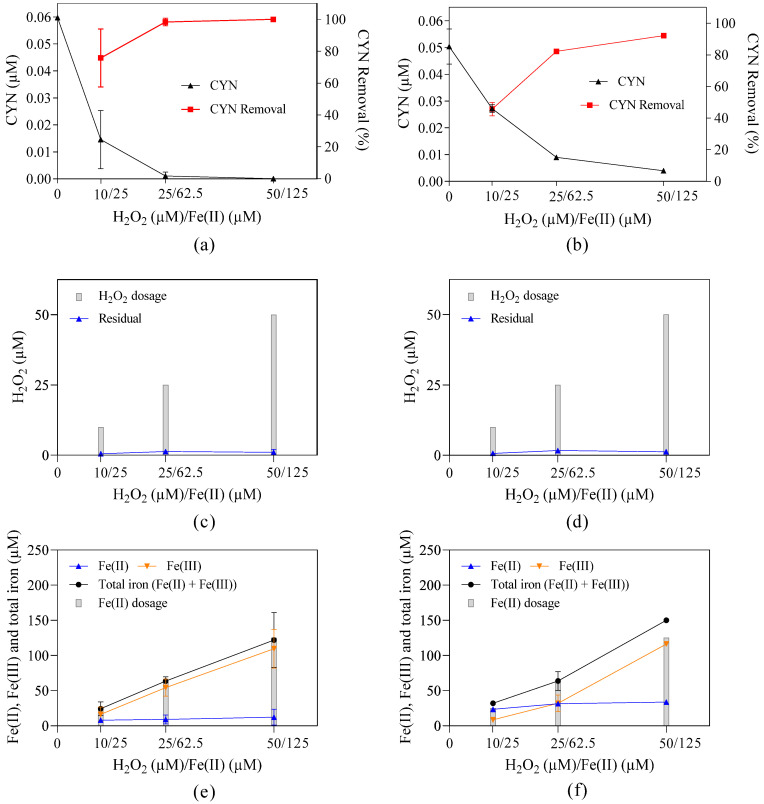
Residual concentration and/or removal of (**a**) CYN, (**c**) H_2_O_2_, (**e**) Fe(II), Fe(III), and total iron in methanol–free ultrapure water, and (**b**) CYN, (**d**) H_2_O_2_, (**f**) Fe(II), Fe(III), and total iron in ultrapure water containing 513.3 µM of methanol. Initial pH about 5.0, H_2_O_2_/Fe(II) molar ratio of 0.4, and 30 min reaction. Error bars represent the standard deviation of the mean based on three replicates.

Following the selection of the value 0.4 as the optimum H_2_O_2_/Fe(II) molar ratio from the studied parameter range, the effect of H_2_O_2_ and Fe(II) dosages was investigated, as shown in Figure 7.

The difference in CYN degradation efficiency increased as the H_2_O_2_ and Fe(II) dosages decreased: for methanol–free ultrapure water and ultrapure water containing 513.3 µM of methanol, CYN degradation efficiency was, respectively, 75.7% and 45.9% with 10 µM H_2_O_2_ and 25 µM Fe(II), 98.3% and 82.2% with 25 µM H_2_O_2_ and 62.5 µM Fe(II), and approximately 100% and 92.1% with 50 µM H_2_O_2_ and 125 µM Fe(II).

Concerning the residual concentrations of the Fenton reagents, it is observed in Figure 7 that the residual concentration of H_2_O_2_ in both subsets of experiments (with and without methanol) was virtually constant, around 1.0 µM. In comparison, the residual concentration of Fe(II) was higher in the subset of experiments conducted in ultrapure water containing 513.3 µM of methanol (between 23.7 and 33.8 µM of Fe(II)) when compared with the subset of experiments conducted in methanol–free ultrapure water (between 8.2 and 12.4 µM of Fe(II)).

This behavior can be attributed to the interaction between methanol, Fenton reagents, and OH•. The OH• has a higher reaction rate constant with methanol, 5.7 to 6.4 × 10^8^ M^−1^ s^−1^ [44,45], in comparison with H_2_O_2_ and Fe(II), 3.3 × 10^7^ M^−1^ s^−1^ and 3.2 × 10^8^ M^−1^ s^−1^ [50], respectively. The excess methanol likely competes with Fenton reagents for OH•, reducing the consumption of Fe(II) and H_2_O_2_ (Equations (1) and (2)), which can promote a higher generation of OH•. Thus, the higher residual concentration of Fe(II) in the subset of experiments conducted in ultrapure water containing 513.3 µM of methanol may indicate a lower consumption of Fe(II) by OH•. Additionally, the lower consumption of Fe(II) by OH• generates less ${OH}^{-}$ (Equation (2)), preventing the increase in pH and Fe(III) precipitation.

Furthermore, under the conditions herein evaluated, the competition between methanol and CYN for OH• slightly decreased the CYN degradation efficiency at the optimum molar ratio of 0.4 from 98.3–100% in methanol–free ultrapure water to 82.2–91.4%. However, a similar behavior was observed for both matrices.

## 3. Conclusions

This study provided evidence of the potential use of the Fenton process to degrade CYN spiked in natural water from Paranoá Lake (Brasília, Federal District, Brazil). The CYN degradation efficiency increased when the H_2_O_2_/Fe(II) molar ratio approximated 0.5, the stoichiometric value for the overall Fenton reaction. The degradation efficiency of CYN also increased when the H_2_O_2_ and Fe(II) dosages increased until reaching a specific concentration, above which any increase in the degradation efficiency was marginal. When the initial CYN concentration increased, the CYN degradation efficiency decreased. The elevation of pH resulted in the reduction of CYN degradation efficiency due to the H_2_O_2_ decomposition and to the alkalinity found in Paranoá Lake water. At the optimum H_2_O_2_/Fe(II) molar ratio, the CYN degradation efficiency was not considerably affected by OH• scavengers, such as NOM, HA, AOM, and methanol. Based on these results, further studies should focus on the feasibility and applicability of the Fenton process in drinking water treatment, emphasizing kinetic analysis, influence on coagulation/flocculation processes, and generation of iron sludge. Moreover, further studies should assess the effects of inorganic ions such as nitrate, sulfate, and chloride on CYN degradation, as these ions may affect the robustness and efficiency of the Fenton process in natural water matrices.

## 4. Materials and Methods

### 4.1. Chemicals

Solid CYN standard (95%) was obtained from Eurofins/Abraxis (Warminster, PA, USA) and used without further purification. Glacial acetic acid (99.7%) was purchased from J.T. Baker (Hexis Científica, Jundiaí, SP, Brazil). Hydroxylamine hydrochloride (96%) and ammonium hydroxide (27% *v*/*v*) were obtained from Synth (Diadema, SP, Brazil). Sodium sulfite (98%), sodium hydroxide (97%), sulfuric acid (98% *v*/*v*), hydrochloric acid (36.5% *v*/*v*), iron (II) sulfate heptahydrate (99%), and iron (III) chloride hexahydrate (97%) were purchased from Dinâmica (Indaiatuba, SP, Brazil). Peroxidase from horseradish (type II), ferrozine (97%), methanol (99.9%), and humic acid sodium salt were acquired from Sigma–Aldrich (São Paulo, SP, Brazil). Hydrogen peroxide (35% *v*/*v*), N,N-diethyl-p-phenylenediamine sulfate salt (98%), sodium phosphate dibasic (98%), sodium phosphate monobasic (98%), and ammonium acetate (97%) were acquired from Neon (Suzano, SP, Brazil).

### 4.2. Cyanobacteria Crude Extract Preparation

The toxic *Raphidiopsis raciborskii* strain (CYP011K) was maintained in the laboratory as a monoalgal and non–axenic culture. The culture was grown under continuous aeration in ASM–1 media [51] with a 12 h photoperiod at 20 °C (room temperature). For the cyanobacteria crude extract containing CYN and its variants, cells harvested during the exponential growth phase were submitted to three freeze–thaw cycles and then sonicated for 30 min at 40 kHz in an ultrasonic bath (USC 5000, Unique, Indaiatuba, SP, Brazil) to break the cells and release the intracellular content. To remove cell debris, the broken cell suspension was filtered in the following sequence: (i) quantitative filter paper, 12.5 cm diameter, cutoff 7.5 µm (3551, Nalgon, São Paulo, SP, Brazil); (ii) fiberglass filter without binder resin, 47 mm diameter, cutoff 0.7 µm (AP4004700, Millipore, Barueri, SP, Brazil); and (iii) mixed cellulose esters membrane, 47 mm diameter, cutoff 0.45 µm (HAWP04700, Millipore, Barueri, SP, Brazil). The crude extract, which contains both cyanotoxins and algogenic organic matter (AOM), was analyzed using a high–performance liquid chromatography coupled with mass spectrometry (LC–MS/MS) method to quantify CYN and was stored at −20 °C until further use.

### 4.3. Paranoá Lake Water

The Paranoá Lake water was obtained from the uptake of the Lago Norte ultrafiltration water treatment plant (Brasília, Federal District, Brazil). It was collected after prefiltration to eliminate large suspended particles. After collection, the measurements of non–purgeable organic carbon, NPOC (Multi N/C 3100, Analytik Jena AG, Jena, TH, Germany), conductivity (Sension 5, Hach, Loveland, CO, USA), alkalinity, apparent color (DR 5000, Hach, Loveland, CO, USA), and turbidity (2100AN, Hach, USA) were performed, respectively, according to the 5310B, 2510B, 2320B, 2120C, 2130B methods described in the Standard Methods for the Examination of Water and Wastewater [52]. Additionally, CYN, pH (Scientific Orion 3 Star portable pH meter, Thermo Fisher Scientific, Waltham, MA, USA), temperature (Sension 5, Hach, Loveland, CO, USA), UV_254_ (DR 5000, Hach, Loveland, CO, USA), H_2_O_2_, Fe(II), Fe(III), and total iron were also measured. The water quality parameters of the Paranoá Lake water are shown in Table 2.

Following the water characterization, the Paranoá Lake water was spiked with aliquots of CYN stock solution to obtain the desired initial CYN concentration, and this solution was referred to as “study water”.

### 4.4. Experimental Setup

The study water and the Fe(II), H_2_O_2_, and sodium sulfite stock solutions were always prepared fresh before the experiments. Unless stated otherwise, the solutions used in the experiments were prepared using ultrapure water (Milli–Q Reference water purification system, C79625, Merck Millipore, Darmstadt, HE, Germany).

For the oxidation experiments, after adjusting the initial pH of the study water, which was monitored during all reaction times, aliquots of H_2_O_2_ and Fe(II) stock solutions were simultaneously added to the study water under vigorous magnetic stirring to initiate Fenton reactions.

When the target reaction time was reached, samples were collected to quantify H_2_O_2_, Fe(II), Fe(III), and total iron. The dissolved fractions of Fe(II), Fe(III), and total iron were obtained by filtering the sample through a 0.22 μm syringe filter (Millex, Millipore, Barueri, SP, Brazil) before analysis. Subsequently, a sodium sulfite solution (2 Na_2_SO_3_: 1 H_2_O_2_) was added to quench the residual H_2_O_2_, thereby halting the production of OH• and other oxidizing agents. After adding the sodium sulfite solution, samples for the quantification of CYN were collected, filtered with a 0.22 μm syringe filter (Millex, Millipore, Barueri, SP, Brazil), and stored at −20 °C until analysis.

Unless indicated otherwise, the experiments were conducted in triplicate using 150 mL of study water (initial CYN concentration of 0.05 µM and initial pH around 5.0) in 250 mL borosilicate glass beakers, with a reaction time of 30 min at room temperature (23 to 25 °C). The pH value of 5.0 was chosen for the experiments to reflect conditions more representative of natural waters, which typically have pH values closer to neutral. All experiments were conducted without light to prevent Fe(III) photoreduction.

Six sets of experiments were performed, as shown in Table 3, to evaluate the degradation of CYN spiked in Paranoá Lake water by the Fenton process.

The study water used in experiments 1, 2, 3, 4, 5.1, and 5.2 was spiked with a stock solution prepared by dissolving 1.2 mmol of CYN standard in 1 mL of methanol to ultrapure water solution (1:1 *v*/*v*). The study water used in experiment 5.3 was spiked with a stock solution of crude extract from *Raphidiopsis raciborskii*. The study water used in experiments 6.1 and 6.2 was spiked with two stock solutions: the first was prepared by dissolving 1.2 mmol of CYN standard in 1 mL of methanol to ultrapure water solution (1:1 *v*/*v*), and the second was prepared by dissolving 1.2 mmol of CYN standard directly in 1 mL of ultrapure water.

Blank study water was utilized to evaluate CYN degradation over time in the absence of Fenton reagents. Furthermore, CYN degradation over time was assessed using H_2_O_2_ and Fe(II) individually, each at the highest concentrations tested in this study: 100 µM for H_2_O_2_ and 375.0 µM for Fe(II).

### 4.5. Analytical Methods

The LC–MS/MS method used for CYN detection and quantification, along with the photometric methods for detecting and quantifying H_2_O_2_, Fe(II), Fe(III), and total iron, have been described in detail previously [27].

The limit of detection of 0.28 nM for CYN, 0.09 µM for H_2_O_2_, 0.20 µM for Fe(II), and 1.26 µM for total iron were determined according to Eurachem guidelines [53]. For CYN quantification, sample extraction and concentration were not required.

## Figures and Tables

**Table 1:** The degradation efficiency of CYN using ultrapure water and Paranoá Lake water as matrices. The experiments were conducted at an initial pH of about 5.0 and a 30 min reaction.

H_2_O_2_ (µM)	Fe(II) (µM)	CYN Degradation Efficiency (%)	
		Ultrapure Water (Ferreira et al. [27])	Paranoá Lake Water (Current Study)
25	62.5	81	66
50	125	91	91.3

**Table 2:** Water quality parameters of Paranoá Lake samples collected after prefiltration at the Lago Norte Water Treatment Plant.

Parameter	Sample 1	Sample 2	Sample 3	Sample 4	Mean (SD)
pH	7.4	7.4	7.5	7.6	7.5 (0.1)
EC (µS/cm)	91.2	93.7	91.7	91.4	92.0 (1.2)
Temperature (°C)	28.3	27.8	26.8	26.3	27.3 (0.9)
Alkalinity (mg/L CaCO_3_)	29	29	28	29	28.8 (0.5)
Apparent Color	4	4	4	4	4 (0)
Turbidity (NTU)	1.69	1.66	1.82	2.21	1.85 (0.25)
UV_254_	0.027	0.027	0.030	0.028	0.028 (0.001)
Fe(II) (µM)	ND	ND	ND	ND	ND
Fe(III) (µM)	1.43	2.50	3.10	1.89	2.23 (0.73)
H_2_O_2_ (µM)	0.09	0.13	0.06	0.04	0.08 (0.04)
NPOC (µM C)	112.4	88.3	84.9	248.9	133.6 (77.8)
CYN (µM)	ND	ND	ND	ND	ND

ND: not detected; SD: standard deviation.

**Table 3:** Summary of the experimental setup: objectives and experimental conditions.

Set of Experiment	Objective	Experimental Conditions	
1	Evaluate the effect of the H_2_O_2_/Fe(II) molar ratio on CYN degradation	H_2_O_2_: 75 µM and Fe(II): 22.1 to 375.0 µM; H_2_O_2_/Fe(II) molar ratio: 0.2, 0.4, 0.5, 0.6, 0.8, 1.0, 1.6, and 3.4; Initial pH: 5.0; CYN: 0.05 µM and Matrix: Paranoá Lake water.	
2	Assess the effect of H_2_O_2_ and Fe(II) dosages on CYN degradation	H_2_O_2_: 25 to 100 µM and Fe(II): 62.5 to 250.0 µM; Optimum H_2_O_2_/Fe(II) molar ratio from set 1; Initial pH: 5.0; CYN: 0.05 µM and Matrix: Paranoá Lake water.	
3	Examine the effect of initial CYN concentration on oxidation efficiency	H_2_O_2_ and Fe(II) concentrations from set 1; Optimum H_2_O_2_/Fe(II) molar ratio from set 1; Initial pH: 5.0; CYN: 0.05, 0.1 and 0.2 µM and Matrix: Paranoá Lake water.	
4	Evaluate the effect of initial pH on CYN degradation	H_2_O_2_ and Fe(II) concentrations from set 1; Optimum H_2_O_2_/Fe(II) molar ratio from set 1; Initial pH values: 3, 4, 5, 7, 9; CYN: 0.05 µM and Matrix: Paranoá Lake water.	
5	Examine the effect of humic acid (HA) and algogenic organic (AOM) matter on CYN degradation	5.1	H_2_O_2_: 25 to 100 µM and Fe(II): 62.5 to 250.0 µM; Optimum H_2_O_2_/Fe(II) molar ratio from set 1; Initial pH: 5.0; CYN: 0.05 µM and Matrix: ultrapure water.
		5.2	H_2_O_2_: 25 to 100 µM and Fe(II): 62.5 to 250.0 µM; Optimum H_2_O_2_/Fe(II) molar ratio from set 1; Initial pH: 5.0; HA concentration: 5 mg/L; CYN: 0.05 µM and Matrix: ultrapure water.
		5.3	H_2_O_2_: 25 to 100 µM and Fe(II): 62.5 to 250.0 µM; Optimum H_2_O_2_/Fe(II) molar ratio from set 1; Initial pH: 5.0; CYN: 0.05 µM from *Raphidiopsis raciborskii* crude extract and Matrix: ultrapure water.
6	Compare the degradation of CYN in methanol–free ultrapure water and ultrapure water containing methanol	6.1	H_2_O_2_: 25 µM and Fe(II): 7.4 to 125.0 µM; H_2_O_2_/Fe(II) molar ratio: 0.2, 0.4, 0.5, 0.6, 0.8, 1.0, 1.6, and 3.4; Methanol: 0 and 513.3 µM; Initial pH: 5.0; CYN: 0.05 µM and Matrix: ultrapure water.
		6.2	H_2_O_2_: 10 to 50 µM and Fe(II): 25 to 125 µM; Optimum H_2_O_2_/Fe(II) molar ratio from set 6.1; Methanol: 0 and 513.3 µM; Initial pH: 5.0; CYN: 0.05 µM and Matrix: ultrapure water.

## Data Availability

The original contributions presented in this study are included in this article and Supplementary Material; further inquiries can be directed to the corresponding author.

## Supplementary Materials

The following supporting information can be downloaded at: https://www.mdpi.com/article/10.3390/toxins16120536/s1, Figure S1: Residual concentrations of (a) H_2_O_2_, (b) dissolved and total fractions of Fe(II), (c) dissolved and total fractions of Fe(III), (d) dissolved and total fractions of total iron, and (e) pH–time profile during Fenton oxidation for different H_2_O_2_ and Fe(II) dosages. Error bars represent the standard deviation of the mean, based on three replicates; Figure S2: Residual concentrations of (a) H_2_O_2_, (b) dissolved and total fractions of Fe(II), (c) dissolved and total fractions of Fe(III), (d) dissolved and total fractions of total iron, and (e) pH–time profile during Fenton oxidation for various initial CYN concentrations. Error bars represent the standard deviation of the mean, based on three replicates; Figure S3: Residual concentrations of (a) H_2_O_2_, (b) dissolved and total fractions of Fe(II), (c) dissolved and total fractions of Fe(III), (d) dissolved and total fractions of total iron, and (e) pH–time profile during Fenton oxidation in matrix ultrapure water containing about 0.05 µM of CYN and 513.3 µM of methanol for different H_2_O_2_ and Fe(II) dosages. Error bars represent the standard deviation of the mean, based on three replicates; Figure S4: Residual concentrations of (a) H_2_O_2_, (b) dissolved and total fractions of Fe(II), (c) dissolved and total fractions of Fe(III), (d) dissolved and total fractions of total iron, and (e) pH–time profile during Fenton oxidation in matrix ultrapure water containing about 0.05 µM of CYN, 513.3 µM of methanol and 5.0 mg/L of AH for different H_2_O_2_ and Fe(II) dosages. Error bars represent the standard deviation of the mean, based on three replicates; Figure S5: Residual concentrations of (a) H_2_O_2_, (b) dissolved and total fractions of Fe(II), (c) dissolved and total fractions of Fe(III), (d) dissolved and total fractions of total iron, and (e) pH–time profile during Fenton oxidation in matrix ultrapure water containing about 0.05 µM and 124.3 µM of AOM for different H_2_O_2_ and Fe(II) dosages. Error bars represent the standard deviation of the mean, based on three replicates.
