# Supplementary material for: Degradation of Cylindrospermopsin Spiked in Natural Water (Paranoá Lake, Brasília, Brazil) by Fenton Process: A Bench–Scale Study
Source: Toxins (Basel). 2024 Dec 12;16(12):536. doi: 10.3390/toxins16120536 (PMC11679458; doi:10.3390/toxins16120536)
Supplement: Supplementary file 1 [file toxins-16-00536-s001.zip › toxins-3301034-supplementary.pdf]

Supplementary material for

Degradation of Cylindrospermopsin Spiked in Natural Water (Paranoá Lake, Brasília, Brazil) by Fenton Process: A Bench–Scale Study

Supplementary material includes 6 pages and 5 figures.

Summary of supplementary material

1. The effect of initial H_2O_2 and Fe(II) concentration on CYN degradation at a fixed H_2O_2/Fe(II) molar ratio 2

2. The effect of initial CYN concentration on the Fenton's efficiency at a fixed H_2O_2/Fe(II) molar ratio 3

3. The effect of humic acid (HA) and algogenic organic matter (AOM) on CYN degradation at a fixed H_2O_2/Fe(II) molar ratio 4

List of Figures

Figure S1. Residual concentrations of (a) H_2O_2, (b) dissolved and total fractions of Fe(II), (c) dissolved and total fractions of Fe(III), (d) dissolved and total fractions of total iron, and (e) pH–time profile during Fenton oxidation for different H_2O_2 and Fe(II) dosages. Error bars represent the standard deviation of the mean, based on three replicates. 2

Figure S2. Residual concentrations of (a) H_2O_2, (b) dissolved and total fractions of Fe(II), (c) dissolved and total fractions of Fe(III), (d) dissolved and total fractions of total iron, and (e) pH–time profile during Fenton oxidation for various initial CYN concentrations. Error bars represent the standard deviation of the mean, based on three replicates. 3

Figure S3. Residual concentrations of (a) H_2O_2, (b) dissolved and total fractions of Fe(II), (c) dissolved and total fractions of Fe(III), (d) dissolved and total fractions of total iron, and (e) pH–time profile during Fenton oxidation in matrix ultrapure water containing about 0.05 μM of CYN and 513.3 μM of methanol for different H_2O_2 and Fe(II) dosages. Error bars represent the standard deviation of the mean, based on three replicates. 4

Figure S4. Residual concentrations of (a) H_2O_2, (b) dissolved and total fractions of Fe(II), (c) dissolved and total fractions of Fe(III), (d) dissolved and total fractions of total iron, and (e) pH–time profile during Fenton oxidation in matrix ultrapure water containing about 0.05 μM of CYN, 513.3 μM of methanol and 5.0 mg/L of AH for different H_2O_2 and Fe(II) dosages. Error bars represent the standard deviation of the mean, based on three replicates. 5

Figure S5. Residual concentrations of (a) H_2O_2, (b) dissolved and total fractions of Fe(II), (c) dissolved and total fractions of Fe(III), (d) dissolved and total fractions of total iron, and (e) pH–time profile during Fenton oxidation in matrix ultrapure water containing about 0.05 μM and 124.3 μM of AOM for different H_2O_2 and Fe(II) dosages. Error bars represent the standard deviation of the mean, based on three replicates. 6

1. The effect of initial H_2O_2 and Fe(II) concentration on CYN degradation at a fixed H_2O_2/Fe(II) molar ratio

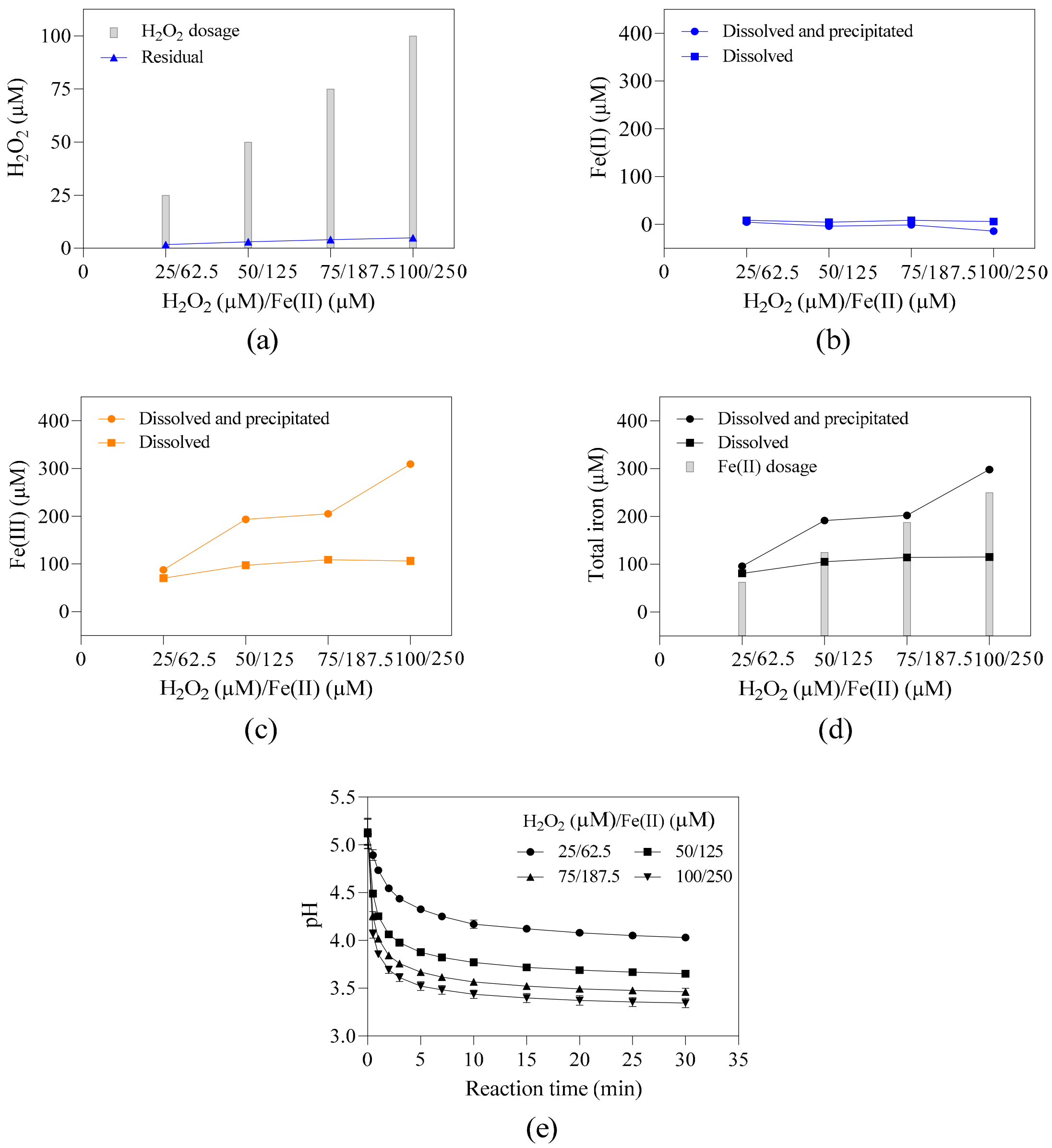

Figure S1. Residual concentrations of (a) H_2O_2, (b) dissolved and total fractions of Fe(II), (c) dissolved and total fractions of Fe(III), (d) dissolved and total fractions of total iron, and (e) pH–time profile during Fenton oxidation for different H_2O_2 and Fe(II) dosages. Error bars represent the standard deviation of the mean, based on three replicates.

2. The effect of initial CYN concentration on the Fenton's efficiency at a fixed H_2O_2/Fe(II) molar ratio

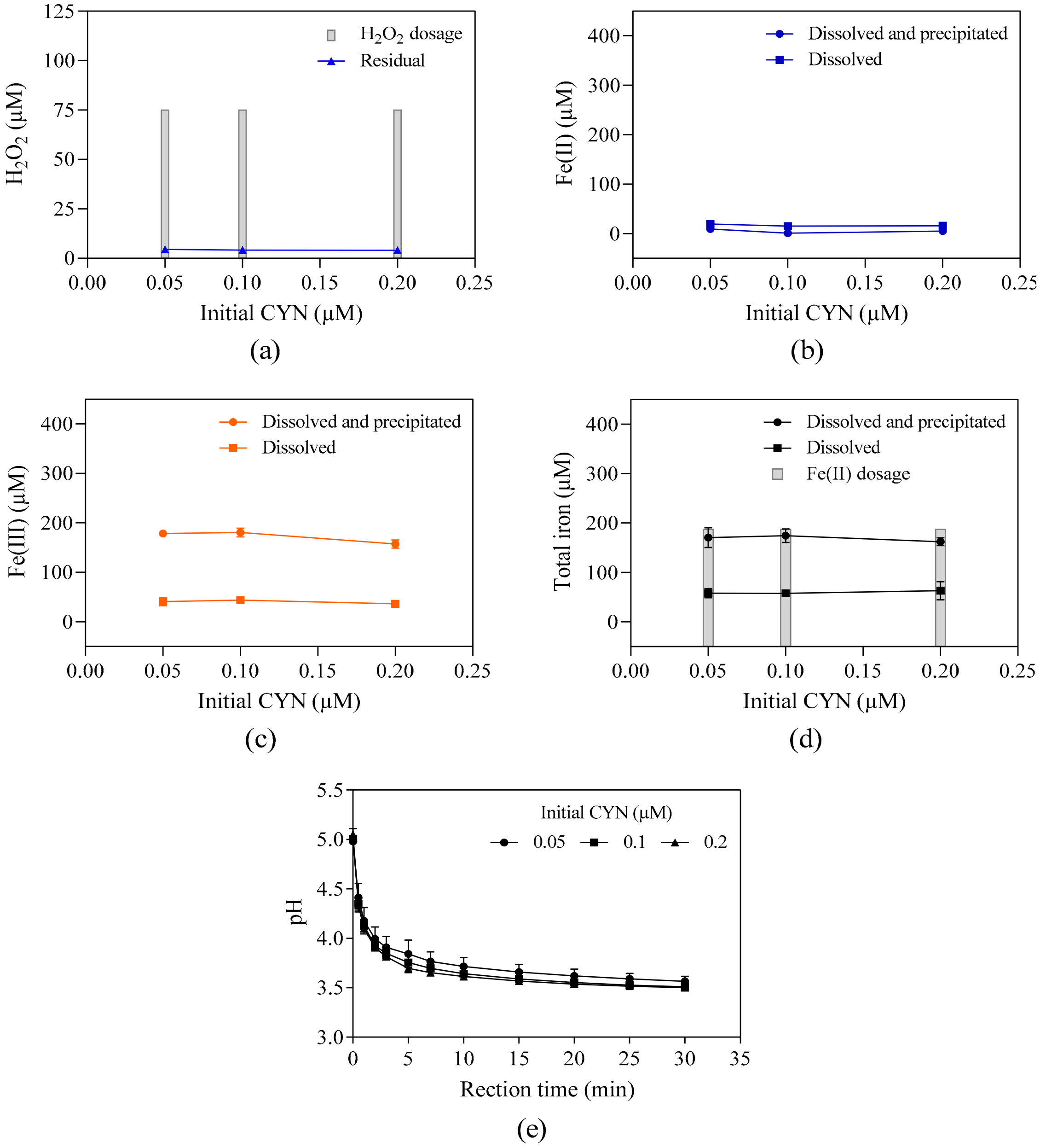

Figure S2. Residual concentrations of (a) H_2O_2, (b) dissolved and total fractions of Fe(II), (c) dissolved and total fractions of Fe(III), (d) dissolved and total fractions of total iron, and (e) pH–time profile during Fenton oxidation for various initial CYN concentrations. Error bars represent the standard deviation of the mean, based on three replicates.

3. The effect of humic acid (HA) and algogenic organic matter (AOM) on CYN degradation at a fixed H_2O_2/Fe(II) molar ratio

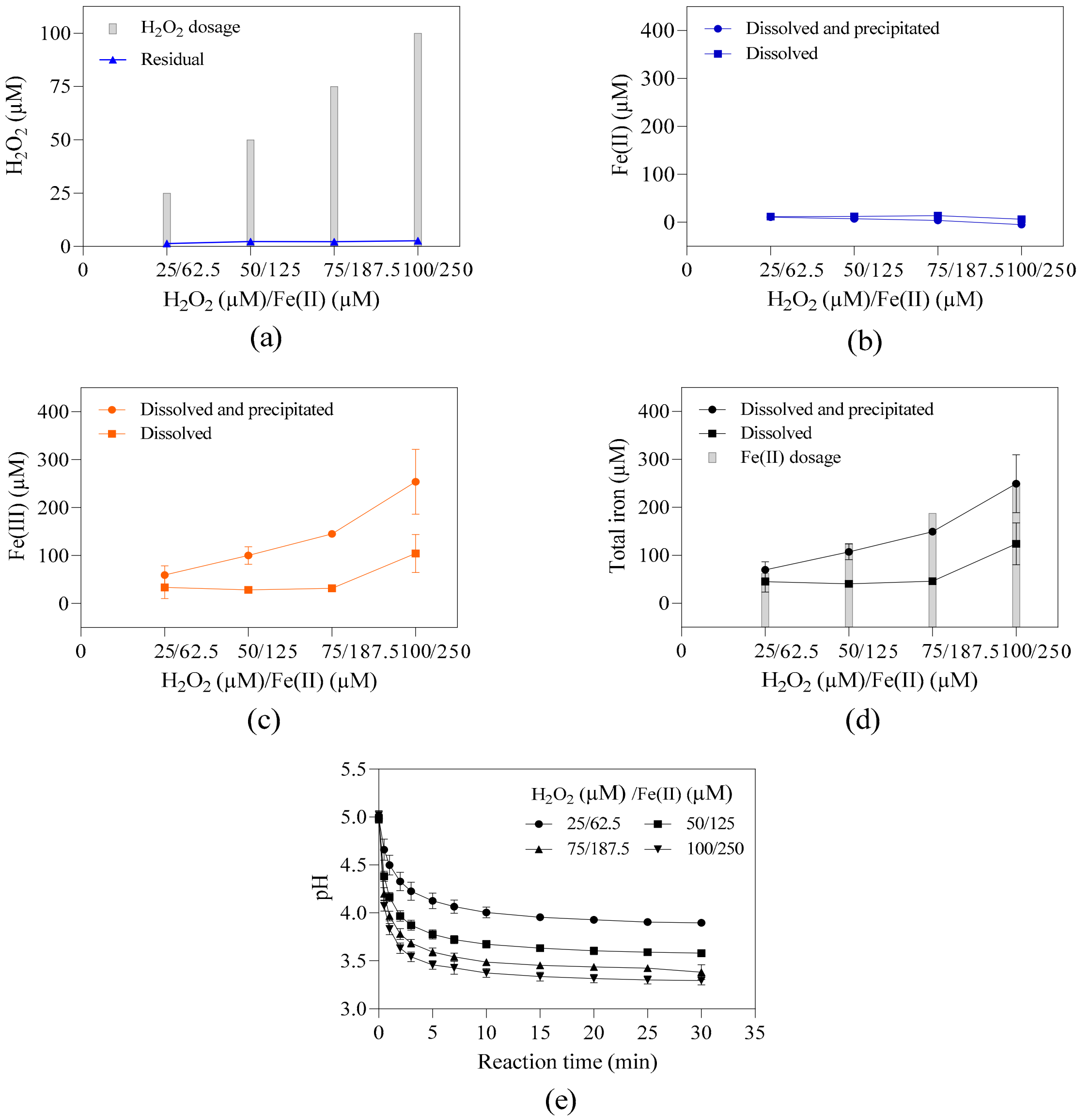

Figure S3. Residual concentrations of (a) H_2O_2, (b) dissolved and total fractions of Fe(II), (c) dissolved and total fractions of Fe(III), (d) dissolved and total fractions of total iron, and (e) pH–time profile during Fenton oxidation in matrix ultrapure water containing about 0.05 μM of CYN and 513.3 μM of methanol for different H_2O_2 and Fe(II) dosages. Error bars represent the standard deviation of the mean, based on three replicates.

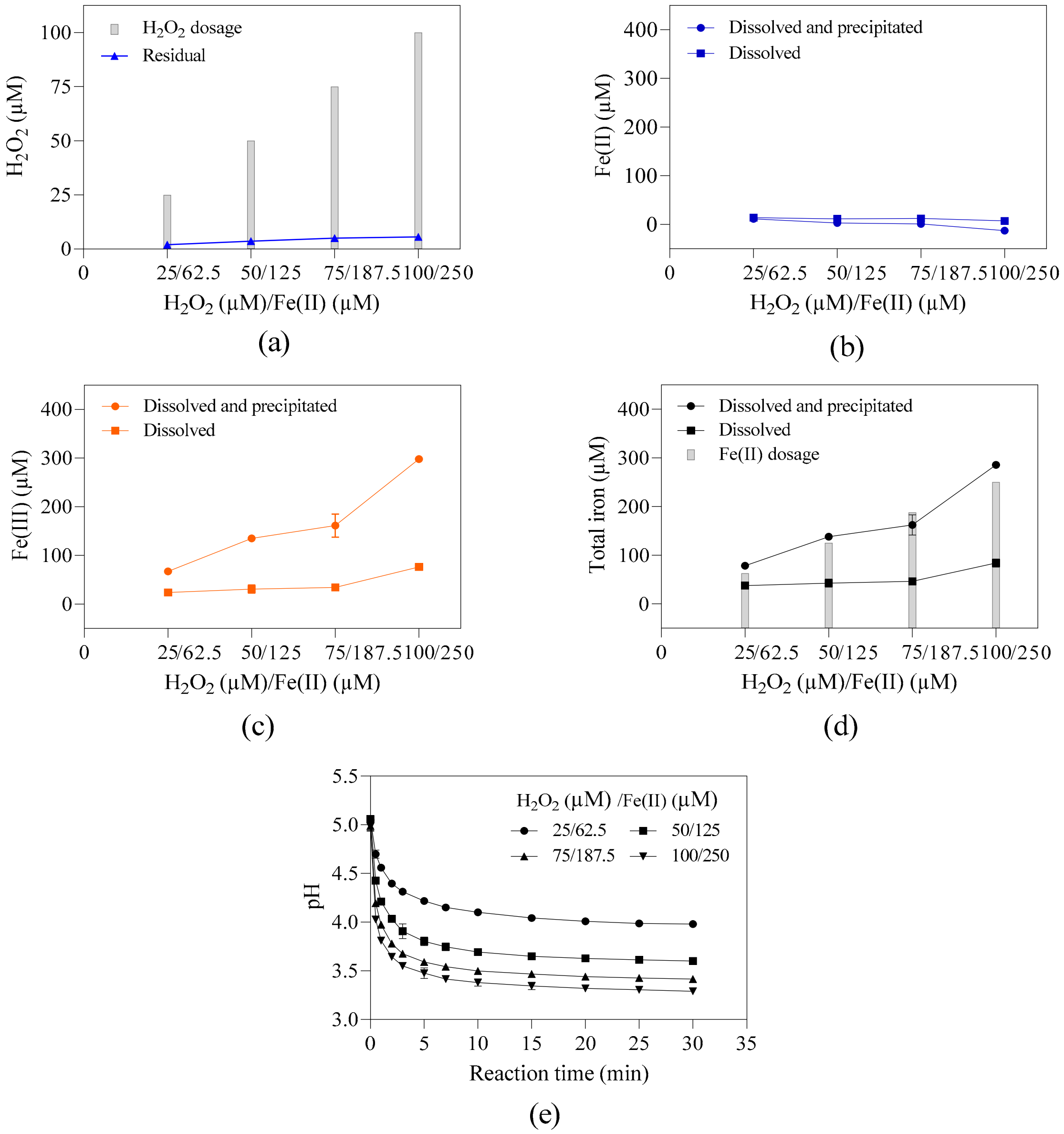

Figure S4. Residual concentrations of (a) H_2O_2, (b) dissolved and total fractions of Fe(II), (c) dissolved and total fractions of Fe(III), (d) dissolved and total fractions of total iron, and (e) pH–time profile during Fenton oxidation in matrix ultrapure water containing about 0.05 μM of CYN, 513.3 μM of methanol and 5.0 mg/L of AH for different H_2O_2 and Fe(II) dosages. Error bars represent the standard deviation of the mean, based on three replicates.

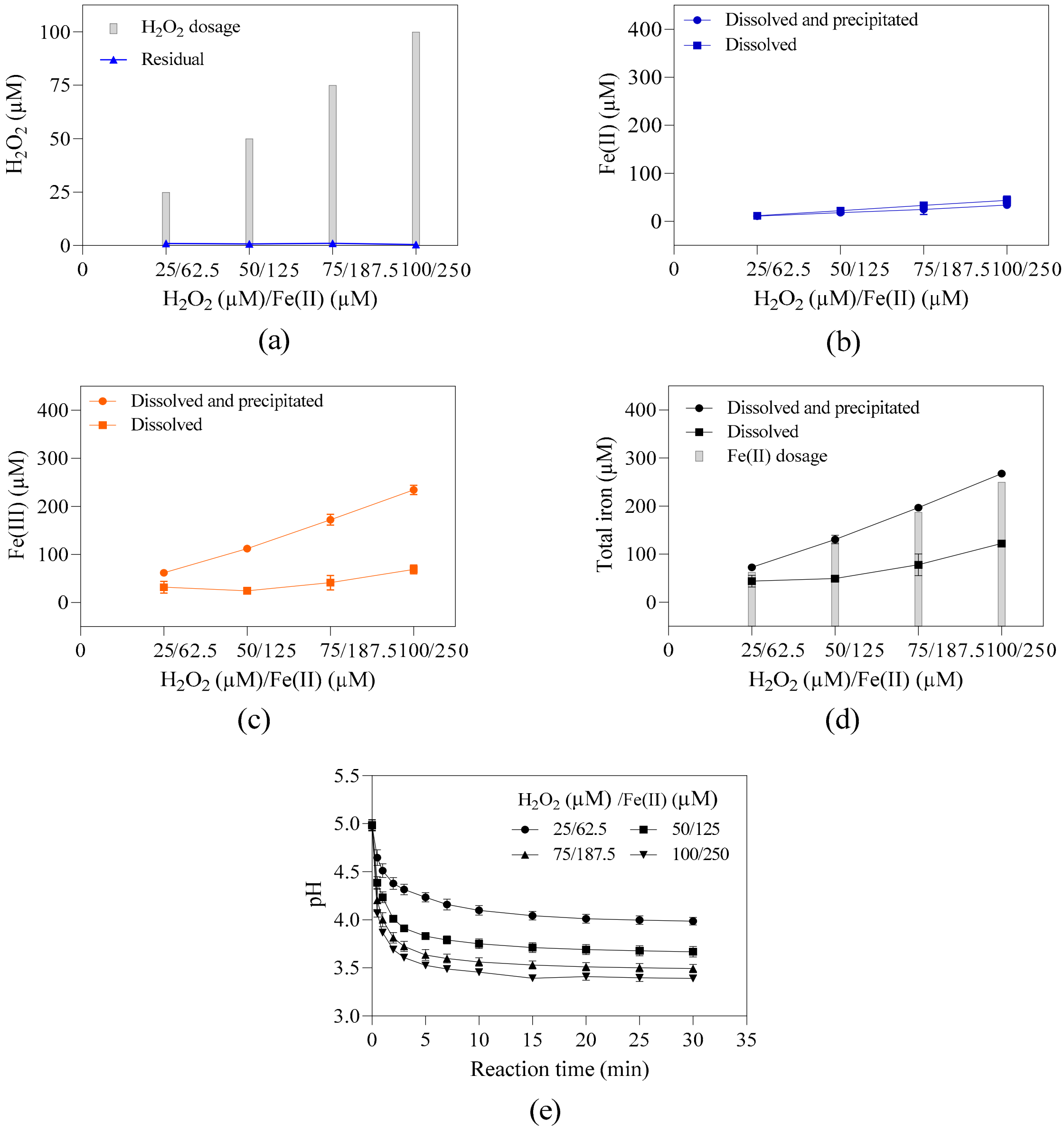

Figure S5. Residual concentrations of (a) H_2O_2, (b) dissolved and total fractions of Fe(II), (c) dissolved and total fractions of Fe(III), (d) dissolved and total fractions of total iron, and (e) pH–time profile during Fenton oxidation in matrix ultrapure water containing about 0.05 μM and 124.3 μM of AOM for different H_2O_2 and Fe(II) dosages. Error bars represent the standard deviation of the mean, based on three replicates.
